# Resource-driven encounters among consumers and implications for the spread of infectious disease

**DOI:** 10.1098/rsif.2017.0555

**Published:** 2017-10-11

**Authors:** Rebecca K. Borchering, Steve E. Bellan, Jason M. Flynn, Juliet R. C. Pulliam, Scott A. McKinley

**Affiliations:** 1 Department of Mathematics, University of Florida, Gainesville, FL, USA; 2 Emerging Pathogens Institute, University of Florida, Gainesville, FL, USA; 3 Department of Biology, University of Florida, Gainesville, FL, USA; 4 Department of Epidemiology and Biostatistics, College of Public Health, University of Georgia, Athens, GA, USA; 5 Center for Ecology of Infectious Diseases, University of Georgia, Athens, GA, USA; 6 Department of Mathematics, Tulane University, New Orleans, LA, USA; 7 South African Centre for Epidemiological Modelling and Analysis, Stellenbosch University, Stellenbosch, South Africa; 8 Department of Mathematics, Tulane University, 6823 St Charles Avenue, New Orleans, LA 70118, USA

**Keywords:** disease ecology, consumer–resource interactions, animal movement, encounter rates, indirect effects, pathogen invasion

## Abstract

Animals share a variety of common resources, which can be a major driver of conspecific encounter rates. In this work, we implement a spatially explicit mathematical model for resource visitation behaviour in order to examine how changes in resource availability can influence the rate of encounters among consumers. Using simulations and asymptotic analysis, we demonstrate that, under a reasonable set of assumptions, the relationship between resource availability and consumer conspecific encounters is not monotonic. We characterize how the maximum encounter rate and associated critical resource density depend on system parameters like consumer density and the maximum distance from which consumers can detect and respond to resources. The assumptions underlying our theoretical model and analysis are motivated by observations of large aggregations of black-backed jackals at carcasses generated by seasonal outbreaks of anthrax among herbivores in Etosha National Park, Namibia. As non-obligate scavengers, black-backed jackals use carcasses as a supplemental food resource when they are available. While jackals do not appear to acquire disease from ingesting anthrax carcasses, changes in their movement patterns in response to changes in carcass abundance do alter jackals' conspecific encounter rate in ways that may affect the transmission dynamics of other diseases, such as rabies. Our theoretical results provide a method to quantify and analyse the hypothesis that the outbreak of a fatal disease among herbivores can potentially facilitate outbreaks of an entirely different disease among jackals. By analysing carcass visitation data, we find support for our model's prediction that the number of conspecific encounters at resource sites decreases with additional increases in resource availability. Whether or not this site-dependent effect translates to an overall decrease in encounters depends, unexpectedly, on the relationship between the maximum distance of detection and the resource density.

## 1. Introduction

The spatio-temporal distribution of resources plays a key role in animal ecology. Studies have examined the impact of naturally occurring resource fluctuations on consumer population dynamics, behaviour and community structure [1–6]. A smaller body of work (reviewed by Becker *et al.* [7] and Sorensen *et al.* [8]) specifically considers the effects that resource provisioning can have for infectious diseases of consumers. Separately, other research has focused on the relationships between resource distribution and animal movement [9–11], and between animal movement and infectious disease [12–16]. However, less attention has been given to the relationship between resource availability and conspecific encounter rates among consumers, despite the clear potential for resource dynamics to indirectly mediate infectious disease transmission via an influence on contact patterns (although see [17,18]).

With this as motivation, we investigate the role resources play in helping to maintain pathogen transmission or facilitate disease emergence. Specifically, we develop a mathematical model for sensing and decision-making by territorial animals that respond to temporarily available, randomly located resources. Through simulations and asymptotic analysis, we characterize the model's major qualitative properties, focusing on the relationship between resource density and consumer encounter rates. To place these results in the context of disease ecology, we then consider the implications of our findings for the ecological system that inspired this work. Namely, we study the potential for a relationship between rabies virus maintenance among a population of black-backed jackals (*Canis mesomelas*) and the annual occurrence of anthrax outbreaks among ungulates in Etosha National Park (ENP) in Namibia [19–21].

**Figure 1.**
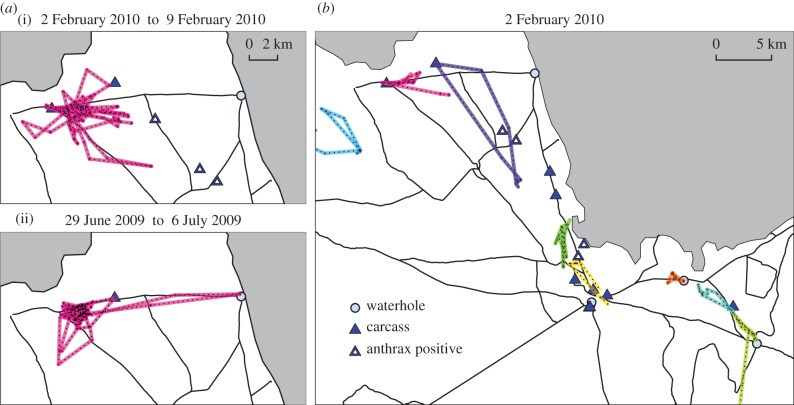
Empirical jackal and carcass location data from Etosha National Park (ENP). (*a*) GPS locations for one collared jackal over one-week time frames, beginning on 2 February 2010 during an anthrax outbreak (i) and 6 June 2009 during the dry season (ii). (*b*) GPS locations for all collared jackals on 2 February 2010. Jackals are differentiated by colour. Each coloured line segment with black dashes connects two GPS fixes for that jackal. Blue circles represent waterholes. Blue triangles indicate locations of known carcasses. White triangle insets indicate that the carcass tested positive for anthrax causing bacteria. Roads are indicated by black lines and the shaded grey areas are part of a salt pan in ENP.

Carcasses that result from seasonal anthrax outbreaks constitute an important supplemental food resource for the jackals. From 2 years worth of jackal and carcass location data [22], we have an improved understanding of how the jackals respond to changes in resource availability. The changes in movement patterns are compelling when considered in the context of the jackal population structure and sociality. Jackals live in territorial family groups consisting of a mated pair, up to five pups, and on occasion a few juveniles [21]. Adults and juveniles regularly hunt and forage (usually alone or in pairs) within and nearby their defendable territory, and opportunistically scavenge on carcasses when they are available. In Zimbabwe, jackals have been directly observed violating territory boundaries in order to feed on carrion; these incursions result in varying degrees of altercation between resident and non-resident jackals [23]. In the location data from Etosha, we observe that jackals sometimes made long treks to visit resources (figure 1; electronic supplementary material, video S1), suggesting that they cross through the territories of neighbouring family groups. Moreover, motion-sensor camera trap recordings at carcass sites captured moments when upwards of 20 jackals convened at a single location at the same time (electronic supplementary material, video S2).

During times of unusually high or low resource availability, jackals may have increased contact with individuals outside their family group [23]. As a consequence, though anthrax bacteria rarely cause disease in carnivores [24–26], an intense uptick in jackal-to-jackal encounters at carcass sites during an anthrax outbreak could lead to an outbreak of a *different* disease in the jackal population [21]. This is an interesting indirect effect: changes in resource availability do not alter the assemblage of attacking pathogens, rather they change the host population's contact frequency in a way that facilitates (or hinders) invasion. While our application is focused on rabies virus, the qualitative results we present also apply to other directly transmitted pathogens such as canine distemper virus or more generally to any pathogen that can be spread through interactions at shared resource sites.

## 2. Model development, data collection and statistical methods

Before developing our models for resource-driven encounters and pathogen invasion, we first provide additional context based on trophic and disease dynamics in our consumer–resource system. As non-obligate scavengers, jackals in Etosha benefit when carcasses are available, but are not threatened with starvation when carcasses are unavailable. Jackals also regularly hunt and forage for sustenance [27]. ENP contains dry savannah habitat, in which migratory herds of zebra, springbok and wildebeest experience seasonal anthrax outbreaks (causal bacterial agent *Bacillus anthracis*) that seem to play a natural regulatory role on their population dynamics. These outbreaks occur during the end of the wet season (March–April) [19,20], with the majority of observed cases occurring in plains zebra (*Equus quagga*; figure 4, inset). *Bacillus anthracis* is a sporulating environmentally transmitted pathogen, with herbivores likely acquiring infection when ingesting contaminated soil or plant material around the site of an old carcass [28]. The mammalian scavenger community that consumes carrion generated by these outbreaks includes lions, hyenas and jackals. These scavengers consume *B. anthracis*-laden carrion without observed disease in ENP [19,21]. While anthrax does not generally cause disease directly in carnivores, it is possible that directly transmitted pathogens, such as rabies virus, may be transmitted between consumers that share a carcass.

Rabies is a highly fatal disease caused by the rabies virus with maintenance populations generally in bat, domestic dog or wild carnivore populations. Rabid carnivores are extremely aggressive and transmit rabies by biting other animals throughout their 5–7 day-long infectious period [23,29], before they eventually die from the disease. Previous work [29–31] has found mixed results concerning whether or not jackals are capable of maintaining rabies transmission without repeated introductions from other host species. Environmental conditions may play a role in determining whether rabies maintenance is possible in jackal populations. In what follows, we expand on this work by informing our pathogen invasion model with results from our mechanistic model for encounter rates at resource sites.

### 2.1. Mathematical model for resource-driven encounters

With the jackal population from ENP in mind, we develop our resource-driven encounters model by first introducing the following general assumptions:

— the locations of both consumers and resources are randomly distributed throughout a spatial region of interest;
— the resources are only available for a given interval of time *τ*_1_ and new resources are located independently of previous ones;
— consumers are territorial, spending most of their time near a home location and have a limited range of detection, characterized by a length scale ℓ;
— consumers prefer to visit the nearest resource they detect;
— they respond to resources independently of other consumers, and
— they are satiated after visiting a resource, and therefore visit at most one resource per unit of time *τ*_2_.

We are interested in the number of conspecific encounters a typical consumer will have as a result of temporarily available resources. Thus, the resources we consider are supplemental in the sense that alternative resources are available and consumer survival does not depend exclusively on the supplemental resource availability.

For the sake of simplicity, and because we believed the choice was reasonable for the jackal population in ENP, we choose the time parameters to be the same, *τ*_1_ = *τ*_2_ = *τ* = one week. We use 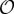 to denote the spatial region we are studying. We reset the supplemental resource landscape each week according to a Poisson spatial process, with intensity parameter *κ*. This means that for any region of area *A* contained in 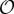, the number of resources in that region is Poisson distributed with mean *κA*. Moreover, if two regions are disjoint, their respective numbers of resources are independent. We assume there is a consumer located at the origin, referred to as the *focal consumer*. The remaining consumers are distributed throughout 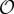 according to a Poisson spatial point process with intensity *ρ*. These intensity parameters correspond to the expected population density produced by the model for the consumers and resources, respectively. In our simulation and mathematical analysis, the size of the landscape is taken to be sufficiently large so that the presence of a boundary does not have an effect on quantities of interest.

**Figure 2.**
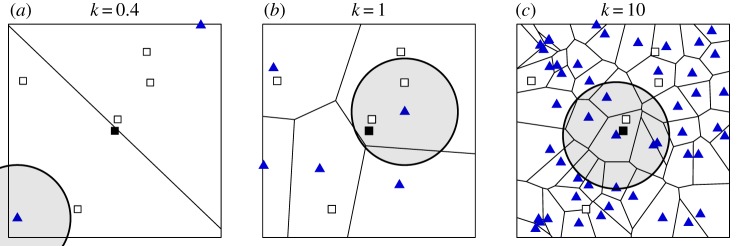
Simulated consumer–resource landscapes with resource intensity *κ*. Voronoi diagrams displaying the regions of ‘attraction’ for each resource. Each blue triangle indicates the location of a resource. The black square and the white squares indicate the locations of the focal consumer and non-focal consumers, respectively. In each panel, there is a grey circle centred at the resource closest to the focal consumer, with a radius representing the detection distance ℓ. The consumers within each circle are close enough to detect the resource closest to the focal consumer. (*a*–*c*) The number of resources displayed in each panel is 2, 5 and 50. (Online version in colour.)

To model the consumer's limited ability to detect resources and/or travel to resources that have been detected, we assume there is a maximum distance ℓ within which a given consumer will detect resources. Moreover, we assume that consumers will detect all resources within a surrounding circle of radius ℓ and will choose to visit the nearest of these detected resources. To understand the consumer–resource landscape, it is helpful to construct Voronoi tessellations of the region 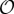 generated using the set of resource locations [32]. Using the R-package deldir [33], we display three such tessellations in figure 2. Each subregion of the tessellation, referred to as a *Voronoi cell*, contains exactly one resource and is comprised of all points that are closer to this local resource than any other. We also refer to a resource's Voronoi cell as its *basin of attraction*. We stress that when resources are rare, the basin of attraction will usually contain many points that are a distance greater than ℓ from the resource. If a consumer is located at such a point, it will not visit any resources during that unit interval of time.

The fundamental goal in the analysis of our model is to understand the number of encounters that occur due to the presence of a particular type of resource. We define the *resource-driven encounter rate* 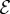 to be the expected number of consumers that choose the same resource as the focal consumer per unit interval of time. In figure 2, the focal consumer encounters 0, 2 and 1 other consumers when visiting its nearest detectable resource. This reveals a fundamental dynamic in the model: that intermediate levels of supplemental resource availability can produce the highest encounter rates. When resources are scarce, resource-driven encounters are rare because it is unlikely that the focal consumer is near enough to a resource to detect it. On the other hand, when resources are common, encounters are rare because nearby consumers have local resources of their own to visit.

To estimate 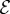 for a given parameter triplet (*ρ*, *κ*, ℓ), we simulated 1000 independent landscapes, calculated the resulting number of encounters for the focal consumer in each, and then took the average of these observations. For most of our simulations, we used the parameter ranges *κ* ∈ (0, 10) and ℓ ∈ (1, 14). As described in appendix A.3, for every triplet (*ρ*, *κ*, ℓ) there is an associated triplet 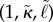 for which 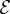 is the same. We, therefore, always set *ρ* = 1 in our simulations and use the transformation 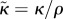 and 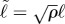 to compute 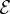 when *ρ* ≠ 1.

### 2.2. Mathematical model for pathogen invasion

To place our encounter rate results in the context of disease ecology, we employ a simple stochastic model of pathogen spillover between two ‘adjacent’ populations. Using terminology from Viana *et al.* [34], we assume that the disease is initially absent in the target population, our main population of interest. In order for any infections to occur, the pathogen must first be introduced into the population. Introduction events take place when an infectious individual from a nearby maintenance population (in which the disease is endemic) infects a susceptible individual in the target population. Owing to our interest in transient seasonal effects, the results are expressed in terms of the duration *T* of the resource increase.

We make three central assumptions:

— the timescale of an outbreak is small relative to the time it takes for significant changes in population size to occur;
— each introduction of a pathogen involves just one initial infectious individual, and
— the arrival times of pathogen spillover events are independent.

Under these assumptions, the initial pathogen invasion process is intrinsically stochastic. Similar to the invasion model proposed by Drury *et al.* [35], we model pathogen introductions as a Poisson process with rate parameter *γ*_spillover_. In other words, we assume that the intervals of time between pathogen introduction events are exponentially distributed and the average time between these events is 1/*γ*_spillover_. For the transmission events among individuals in the target population, we use a stochastic susceptible–infectious–susceptible (SIS) model. Because the total population size is fixed in this model, it is only necessary to track the state transitions for the infectious group, whose population size at time *t* is denoted *I*(*t*). The transition rates for our continuous-time Markov chain (CTMC) are given by

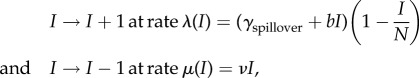
where *N* is the target population size, *ν* is the clearance (or disease-related mortality) rate and *b*(1 − *I*/*N*) is the average number of transmissions per unit interval of time by an infectious individual when the infectious population has size *I*.

While this simple model of transmission ignores other potentially relevant characteristics (e.g. latent periods, population turnover and acquired immunity), our present focus is on how consumer–resource interactions modulate transmission dynamics in the early introduction phase. We are specifically interested in the probability that the level of infection can reach an endemic state in the target population before the period of resource increase dissipates. Given our context that the disease dynamics take place over a large area and the pathogen introductions are relatively rare, we introduce a fourth assumption:

— each pathogen introduction resolves itself independently in the target population (either to extinction or invasion).

Mathematically, this is tantamount to omitting the *γ*_spillover_ term in the transition rate formulae and treating each pathogen introduction event independently. The ‘endemic equilibrium’ is the minimum size for the infectious population such that the rate of increase is less than or equal to the rate of decrease. We consider a pathogen introduction to be ‘successful’ if the size of the resulting infectious population eventually exceeds the endemic equilibrium value:
2.1
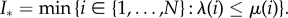
We then study the continuous-time Markov chain {*I*(*t*)}_*t*≥0_ with the transition rates

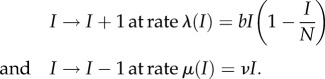


In a sense made rigorous by Kurtz [36], when *N* is large this stochastic system behaves more and more like an associated ordinary differential equation (ODE),
2.2
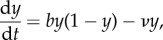
where we interpret *y*(*t*) as the proportion of the population that is infectious at time *t*. If *b* > *ν* and *y*(0) > 0, then *y*(*t*) converges to the equilibrium value y_*_ = 1 − *ν*/*b*. Otherwise *y*(*t*) → 0 as *t* → ∞. Following the terminology used by Ball [37] (see also Heffernan *et al.* [38]), we refer to 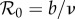 as the *reproductive ratio*.

**Figure 3.**
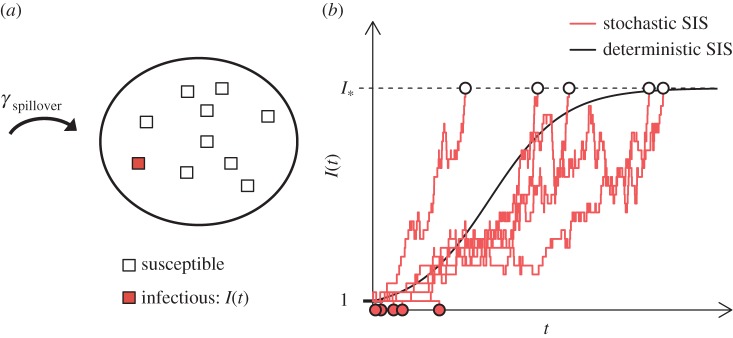
Pathogen invasion model and simulations. (*a*) Pathogen introductions (index infections) occur at a rate *γ*_spillover_. (*b*) Number of infectious individuals resulting from the introduction of one infectious individual in a population of size *N* = 50. Ten sample paths for the stochastic SIS model defined in §2.2 are plotted (red lines) with the solution to the analogous ordinary differential equation model (black curve). Our representation for having achieved the endemic state is I_*_ (dashed horizontal black line), which is defined in equation (2.1). Open circles are plotted when I_*_ is reached before 0 and red circles indicate when the pathogen died out of the population before reaching I_*_. (Online version in colour.)

In contrast with the ODE model, no matter how large *N* is, in the stochastic model there is always a chance that an infectious lineage will go extinct before it reaches an endemic state. In figure 3, we display 10 stochastic SIS paths with a population size of 50 with *b* = 2 and *ν* = 1. Some of these paths quickly go extinct, while others reach the endemic state. Overlaid on the stochastic paths is *Ny*(*t*), the rescaled solution to the associated ODE (2.2), with initial condition *Ny*(0) = 1. In appendix B, we review how to approximate the probability of successful invasion assuming that a pathogen has been introduced at time zero:

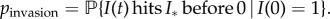

To connect the resource-driven encounter rate, 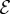, to the pathogen-transmission model, we first note that not all encounters involving infectious and susceptible individuals lead to a new infection. For example, in our model, a resource-driven encounter is defined to occur if two individuals visit the same resource site in the same week, but this does not mean they visit concurrently. Even if they visit concurrently this does not ensure pathogen transmission. We define *p*_inf_ to be the probability that a resource-driven encounter results in transmission. Then our expected number of new infections arising from a single infectious individual is 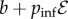 and the corresponding reproductive ratio is
2.3
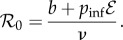
Since our focus is on the effects of resource abundance on encounter rates, we will primarily consider changes driven by seasonal resource density. We, therefore, will consider a time-dependent version of the basic reproduction ratio, which we denote 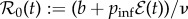, and investigate if and when 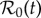 is greater than one and how its value changes throughout the year. Changes in 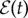 can be driven by changes in any of the resource-driven encounter model parameters (i.e. consumer density, resource density or detection distance).

### 2.3. Model application to jackals in Etosha National Park

*Data collection.* Jackals were captured from January 2009 to July 2010 in central ENP as part of a larger study on jackal movement and anthrax ecology (Bellan *et al.* [21]). Twenty-two adult jackals were fitted with GPS (global positioning system; African Wildlife Tracking, Pretoria, Republic of South Africa) collars based on the requirement that they were large enough to limit the collar to less than 6% of the animal's body weight. Movement data were acquired from collars by VHF radio-tracking animals and downloading recorded hourly GPS fixes with UHF download. Owing to challenges associated with acquiring downloads, there is some variation in the time intervals between recorded locations. In some cases, there are missing data points; and in a few cases, observations were made more frequently than once per hour. The duration of time each collared animal was observed also varied greatly, from a few weeks to 2 years, for a total of 13.5 jackal-years of (roughly) hourly location data.

**Figure 4.**
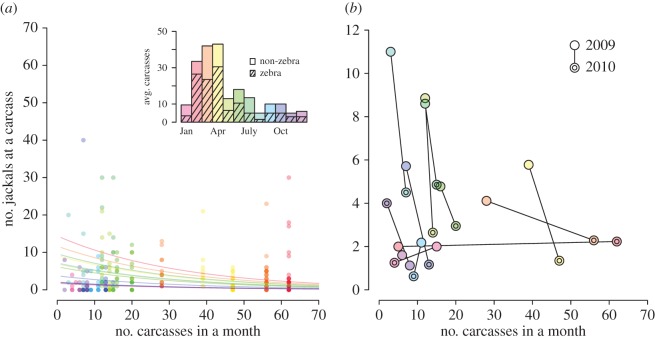
Empirical monthly carcass availability and jackal visitation to carcass sites data from ENP. (*a*) For each month–year pair, the number of observed carcasses is counted (*x*-axis). We plot the number of jackals recorded at each carcass versus the number of observed carcasses in the corresponding month. Jackal counts at carcasses are colour-coded by month of the year as indicated by the bar chart in the upper right corner. Points are shaded so that darker shading indicates more observations. Regression lines are plotted for each month in the corresponding colour. Inset: The monthly average number of observed total carcasses (solid bars) and zebra carcasses (striped bars). (*b*) For each month–year pair, the average number of jackals observed at carcasses is plotted. Repeated months are connected by lines.

ENP staff and, as part of an intensive study on anthrax ecology, other researchers routinely record carcasses observed throughout the park. Multiple characteristics of a carcass are recorded, such as: species, date of observation, level of degradation and cause of death. During the jackal movement study (January 2009 to November 2010), jackal counts were recorded for 299 of 411 carcass sites (178 of 244 zebra carcass sites). These data are displayed in figure 4. Additionally, motion-sensor camera traps (Reconyx RC55) were deployed at 31 fresh zebra carcasses to study how scavenger activity and carcass decomposition processes evolve over time after an animal's death (see [39] and electronic supplementary material, video S2). Cameras were programmed to capture at a rate of 1 frame per second for 10 s after a motion trigger with a 60-s delay between when they could be triggered again, allowing semi-continuous recording of activity for up to approximately three weeks post-death.

*Identifying resource visits.* For each recorded instance of a carcass, we assigned a ‘carcass active interval’ based on its estimated time of death and the level of degradation at the time of discovery (if recorded). This window lasted up to 6 days. Six days also served as the baseline duration of availability, used when low or no level of degradation was recorded. For each jackal that was tracked in the park contemporaneously with a known carcass, we computed a ‘time-local average position’, i.e. the mean of all recorded positions of the jackal during the carcass active interval. The distance between this average position and the location of the active carcass was assigned to be the distance of a resource visit or non-visit. If the jackal's minimum distance from the carcass of interest during this period was less than 100 m, we classified the event as a resource visit. We chose this distance to account for jackal locations between the hourly GPS fixes. We also note that this distance is small relative to the study area in which the jackals move and corresponds roughly with the side length of the triangles depicting resource locations in figure 1.

Furthermore, we justify our classification of a ‘resource visit’ by performing a randomization test that confirms that carcass presence affects whether a visit or non-visit is recorded. For example, if our choice of observed distance between jackal and carcass was too large, we might record visits just as a result of typical day-to-day jackal movement, rather than actual directed movement to the carcass. We ran our visit identification algorithm on 1000 sets of randomized carcass locations. For each site, we held the time that the site was available constant and reassigned the location by sampling (without replacement) from the recorded carcass locations. For each permuted dataset, we then calculated the number of ‘resource visits’ in the same way as described above for the observed data. Using the true carcass location data there were 10 and 44 visits from jackals with a time-local average of 10+ km and 5+ km, respectively, from the carcass locations. Out of 1000 sets of randomized carcass locations, the maximum number of 10+ km and 5+ km visits was 6 and 15, corresponding to a *p* < 0.001 for both distances, and indicating that there is a highly significant association between jackal locations and identified carcass sites.

*Statistical data analysis of resource visitation response.* We challenged the predictions of our resource-driven encounter model by examining how the number of jackals observed at carcass sites varied with carcass density in ENP using multivariate regression. To account for over-dispersion in the jackal counts at carcass sites data, we fit a negative binomial generalized linear model with the number of observed carcasses as a predictor variable and the number of jackals observed at the carcass site as the response variable (available from January 2009 to November 2010). We also included predictor variables for each month of the year to allow for variation in environmental effects (e.g. wet/dry season), population processes (birth pulses, dispersal, etc.), variation driven by turnover in researchers present in the field, and the daily activity patterns of researchers. To be precise, let *y*_*i*_ be the response variable for the number of jackals observed at a carcass when there are *i* carcasses. Then
2.4
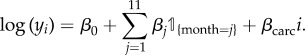
For example, in an April with *i* total carcasses, the expected number of visitors observed at a carcass would be 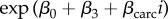.

## 3. Results

By way of simulation and analysis, we are able to characterize the most prominent qualitative features of the expected number of encounters experienced by a focal consumer, as they depend on the expected resource density *κ* and the maximum distance of detection ℓ. While we report the results of the specific model described in the previous section, as long as a model is consistent with the listed assumptions, then our fundamental conclusions are the same: there is a non-monotonic relationship between the expected resource-driven encounter rate and the resource density; the maximum potential encounter rate can be large in terms of its impact on the critical disease ecology parameter 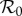; and, somewhat surprisingly, low resource densities are associated with the largest encounter rates.

### 3.1. Analysis of the consumer–resource model for encounter rates

We summarize the emergent dynamics of our mathematical model as follows. For fixed values of *ρ* and ℓ:

— *From the point of view of an available resource (carcass), the number of visitors decreases with κ*. The presence of additional resources increases the number of options for consumers and so, as *κ* increases, the expected number of visitors at a given resource site decreases.
— *From the point of view of an individual consumer, the number of encounters increases, then decreases with κ*. When resources are scarce, most consumers will not be close enough to detect them. Increasing *κ* means that more and more consumers visit resources, leading to increased consumer–consumer interactions. The effect is not monotonic though. When resources are abundant, consumers will generally detect more of them. Owing to this increase in available options, it becomes less likely that multiple consumers will visit the same resource.

From the point of view of a given resource site, there are two limiting factors on the number of visitors: (i) the size of the resource's basin of attraction, as defined by the Voronoi tesselation described in §2.1 and presented in figure 2; and (ii) the consumers' limited distance of detection. As *κ* increases, the resource's basin of attraction decreases in size, therefore, limiting the pool of consumers that would choose it. In §3.2, we present an analysis of the ENP data, wherein we find some physical evidence that the number of jackals expected at a particular carcass decreases with the number of carcasses available at the time.

**Figure 5.**
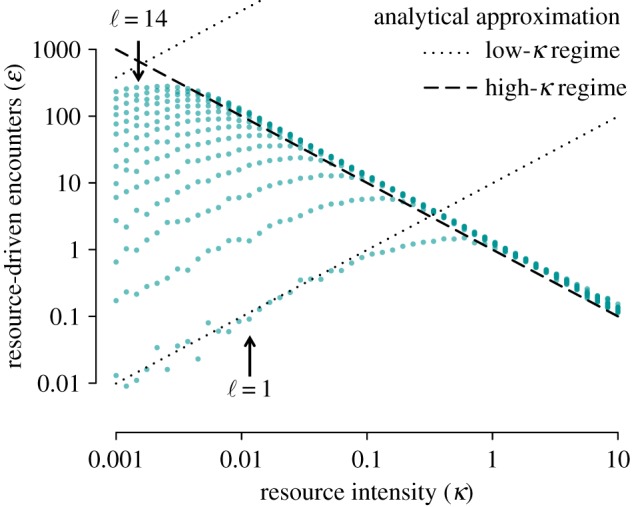
Encounter rate model simulations and predictions. Each dot represents the average number of resource-driven encounters for a given resource intensity (*κ*) and distance of detection (ℓ). To calculate the average we found the model results for over 1000 simulated resource landscapes. Results are displayed for detection distances ranging from ℓ = 1 to ℓ = 14. The simulations agree well with the theoretical model predictions described in §3.1 and derived in appendix A. The dotted and dashed lines indicate the theoretical model predictions for low- and high-resource density regimes, respectively. The intersection of the dotted and dashed lines is the order-of-magnitude estimate for the peak encounter rate and associated critical resource density described in §3.1. (Online version in colour.)

**Figure 6.**
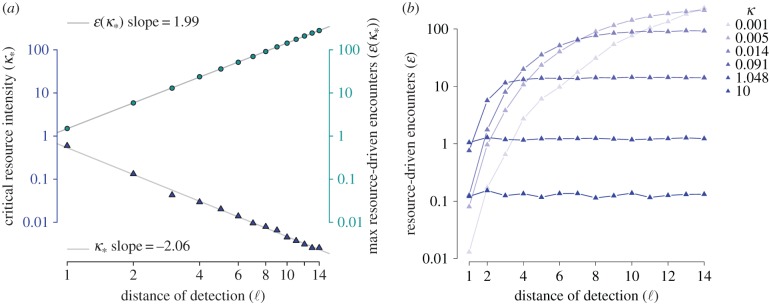
Encounter rate model simulations and characteristics. (*a*) For each detection distance ℓ (bottom axis), the maximum number of encounters from simulations over resource densities *κ* between 0.01 and 100 are plotted as teal circles (right axis). The corresponding critical resource densities, κ_*_ values (left axis), where the encounter rate is maximized, are plotted as blue triangles. (*b*) Simulated number of resource-driven encounters as a function of distance of detection. Lighter-to-darker shading corresponds to increasing values of the resource density *κ*.

From the consumer point of view, a focal consumer is always in the basin of attraction of some resource (figure 2); however, when resources are scarce it is unlikely that it will be close enough to detect the nearest resource. On the other hand, when resources are abundant, the area of the basin of attraction can be very small compared to the focal consumer's detection area, limiting the pool of potential consumers that might share the resource. In figures 5 and 6, we provide a comprehensive view of the dependence of a focal consumer's encounter rate 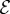 on resource density. In appendix A, we provide the details of our mathematical analysis of the model and rigorously demonstrate certain prominent features of the relationship between encounters and resource availability: namely, the asymptotic power law in both the scarce and abundant resource regimes, as well as in the small and large distance of detection extremes. Furthermore, we provide an approximate formula for the resource density κ_*_ that leads to the maximum number of encounters for a given distance of detection and consumer density.

*Asymptotic results.* In figure 5 we see that 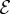, the expected number of encounters for the focal consumer, exhibits power law behaviour in both the small- and large-*κ* regimes. Regardless of the value of ℓ, all of the encounter rate curves overlap in the large-*κ* regime. For small *κ*, the log–log slope is the same for all ℓ, but the leading coefficient differs. In theorem A.1, we demonstrate that when resources are scarce (small *κ*), the resource-encounter function is asymptotically linear in *κ*. Furthermore, we are able to establish the leading coefficient, yielding the *small-κ* approximation 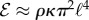, which is also validated by simulation. For example, in figure 5, the black dotted line is the small-*κ* approximation when ℓ = 1, which agrees well with simulations (teal dots) for *κ* < 0.1. It is not possible to derive a rigorous estimate for the large-*κ* regime because the analysis reduces to a major unsolved problem in spatial point process theory (see paragraph preceding theorem A.5). Nevertheless, we argue that 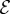 scales with *κ*^−1^ in the abundant resource limit. Following the discussion in appendix A.3, we present the *large-κ* approximation 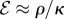 (figure 3, black dashed line). The correct leading coefficient appears to be larger than *ρ*, but we were unable to obtain the exact value by mathematical analysis.

*Characterizing the encounter rate peak.* For reasons discussed in §3.4, perhaps the most important ‘landmark’ of the resource-encounter function is its peak. Unfortunately, it is difficult to directly analyse the magnitude of the peak and the corresponding critical resource density. However, there is a natural first-order estimate that involves the small- and large-*κ* approximations. Solving for their intersection yields the estimate 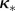 ≈ (1/*π*)ℓ^−2^ and 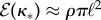, where κ_*_ is the resource intensity that leads to the maximum resource-driven encounter rate. From figure 5, it is clear that this is an overestimate, but not dramatically so. Using 1000 simulations at an array of *κ* and ℓ values, we found the following estimates using a linear regression (depicted as grey lines in figure 6*a*)
3.1
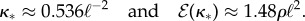
While the exponents align well with those found by looking at the intersection of the small- and large-*κ* approximations, we were unable to obtain a satisfactory explanation of the leading coefficients through direct mathematical analysis.

*Dependence on distance of detection.* In addition to characterizing the encounter rate's dependence on *κ*, we are also able to obtain an understanding of the asymptotic dependence of the resource-driven encounter rate on the maximum distance of detection parameter ℓ. As ℓ → 0, the encounter rate function behaves like ℓ^4^ (theorem A.1). As might be expected, this function is monotonically increasing in ℓ and saturates to a limiting value for large ℓ (theorem A.5). The corresponding simulation results are displayed in the right panel of figure 6. The limiting value corresponds to the expected area of the basin of attraction in which the focal consumer resides. As mentioned earlier, this exact value is not known, but it scales like *κ*^−1^, which is why the limiting values in the right panel of figure 6 are largest for the smallest values of *κ*.

*Reducing the dimension of the parameter space.* Though there are three parameters in the mathematical model, we found that there are truly only two degrees of freedom in the parameter space. As shown in appendix A.3, for every triplet (*ρ*, *κ*, ℓ), there is a corresponding triplet 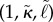, where 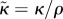 and 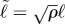, such that the expected numbers of encounters for the focal consumers are the same, i.e.

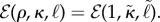
Notably, 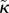 and 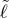 are non-dimensional quantities and all formulae introduced in the previous section can be expressed using them

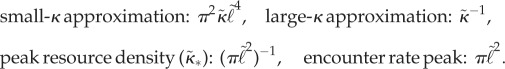
Both non-dimensional quantities have informative biological interpretations. While it is straightforward to understand the significance of 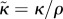 (the ratio of the resource density to the consumer density), the meaning of 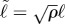 is more subtle. If we imagine breaking up the landscape into territories of equal size, then each consumer would be allocated a region of area 1/*ρ*. In order to relate territory *area* to detection *distance*, we define a characteristic territory length. One natural way to define the territory length scale is the square root of the area, in this case 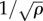. If the regions are square, then 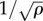 would be the length of each side. We view 
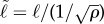
 as the ratio of the distance of detection to the typical length of a consumer's space allocation. Visually, this amounts to dividing the detection distance into territory length intervals. Thus, in this simplified setting, 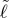 is roughly the number of defendable (i.e. non-overlapping) territories a consumer is willing to cross in order to visit a resource.

### 3.2. Validation of a model prediction: visitation at resource sites decreases with resource density

The mathematical model makes predictions about both full-population scale encounter rates and local single-resource site encounters. For the latter, from the perspective of a given carcass site, the model predicts that the maximum number of visitors should be observed when the resource density is the lowest. This is because in the sparse resource-density regime there is little to no competition for consumers. As the resource-density increases, the expected number of visitors should decrease. We consulted the ENP dataset to investigate whether this effect can be observed for jackals and their tendency to visit carcasses that seasonally vary in abundance.

In the study area [21], the number of carcasses available for jackal scavenging varies seasonally (figure 4*a* inset). Between February and April, there is a resource pulse resulting from annual anthrax outbreaks in the local ungulate population. These outbreaks occur during the end of the wet season [19,20]. The timing and severity of anthrax outbreaks appears to be different between 2009 and 2010. The difference in severity provides an opportunity to make comparisons between the same months of the year but with very different numbers of available carcasses. In March and April, for example, the average number of jackals observed at carcasses decreases markedly from 2009 to 2010 when there are more carcasses available. In fact, for eight of the 11 months where pairwise comparisons are possible, the average number of jackals observed at a carcass decreased when more carcasses were available in that month (figure 4*b*).

In our negative binomial generalized linear model, we found a significant negative correlation between the number of observed carcasses and the observed number of jackals at a carcass (*β*_carc_ =−0.030, 95% CI: [−0.043, −0.018]). This result is consistent with our model prediction that at medium to high resource densities, the expected number of resource-driven encounters decreases with carcass density.

### 3.3. Model parametrization using Etosha National Park data

Our estimate of the resource density, *κ*, is based on carcass surveillance data from the ENP during 2009 and 2010 [22]. The average number of carcasses recorded each month (figure 4*a*) was divided by four to get a weekly number of carcasses available. Since not all carcasses are observed, we followed Bellan *et al.* [39] in multiplying by a scaling factor of four to account for expected unobserved carcasses. We then divided the expected weekly number of carcasses available in each month by the area of the study region from Bellan *et al.* [21], approximately 1000 km^2^. This area contains all of the locations where carcasses were observed and jackal positions recorded. The resulting *κ* estimates ranged from 0.005 km^−2^ (August and November) to 0.043 km^−2^ (April).

As suggested by the non-dimensionalization argument above, we interpret *ρ* as the density of defendable jackal territories. Non-overlapping jackal territories in ENP were estimated to be between 4 and 12 km^2^. This is comparable to estimates that were made for jackal populations in coastal Namibia (0.2–11.11 km^2^ [40]) and South Africa (3.4–21.5 km^2^ [41]). Noting the observation from Bellan *et al.* that jackals are ‘unusually dense’ in ENP [21], we set the typical jackal territory size to be 5 km^2^, so that *ρ* = 0.2 km^−2^.

The interpretation of the parameter ℓ from the data requires some discussion. In the mathematical model, ℓ is the maximum distance at which a consumer can detect and then respond to a resource. We can think of the model as assuming that the probability of detecting a resource is one within a distance ℓ and zero outside that distance. Of course, in reality, this detection probability likely decreases steadily as a function of distance. The range of detection also likely depends on environmental conditions (e.g. surrounding vegetation density) and specific characteristics of individuals (e.g. age and sex) that are beyond the scope of our model. Rather than identify a specific value that we definitively claim to be the best estimate of ℓ, we used the jackal movement data to find a range of reasonable values and then focused our investigation on two values that showcase the contrasting regimes predicted by our model.

**Figure 7.**
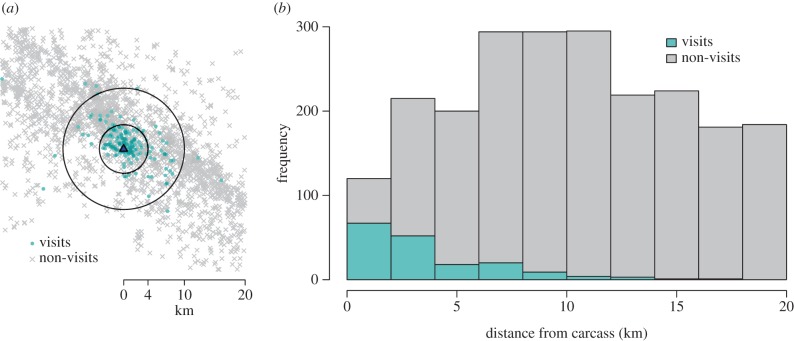
Carcass visitation data based on empirical jackal and carcass location data from ENP. For each recorded carcass, contemporaneously collared jackals were identified as either visiting or not visiting the carcass using the methodology described in §2.3. (*a*) Relative positions of jackals plotted with respect to the location of each known carcass. Jackal locations were calculated as the average of their GPS fixes that occurred during the ‘carcass active interval’ described in §2.3. (*b*) Stacked histogram for the times that jackals chose to visit a carcass (teal bars) and the times that jackals refrained from visiting a carcass (grey bars).

**Figure 8.**
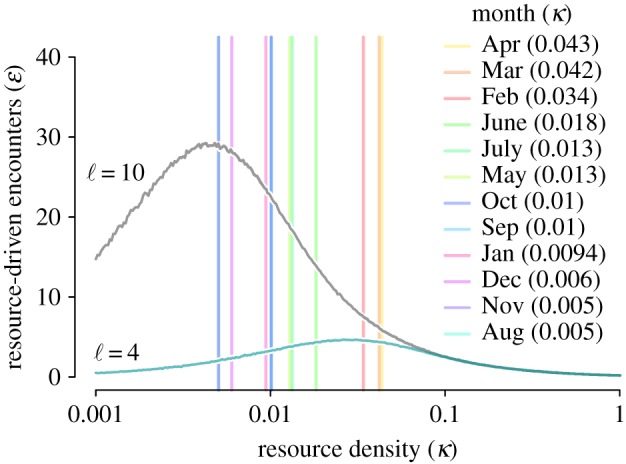
Encounter rate model simulations in relation to empirical carcass density estimates from ENP. Simulated number of resource-driven encounters for two choices of the detection distance parameter over a range of resource intensities. The vertical lines indicate the estimated month-by-month carcass densities observed in the ENP dataset (see figure 4 inset for corresponding carcass counts).

In figure 7, we display a scatter plot of all jackal average positions relative to known carcasses and mark each with a teal dot or a grey x depending on whether the jackal visited the carcass or not. Jackals were observed to visit known carcass sites as far as 15 km away, but a large majority of carcasses visited were in a range of 0–4 km. As expected, the probability that a jackal visited a resource decreased with distance, but it is not known whether this was because the jackals were not aware of more distant carcasses, or because there were other carcasses or alternate resources nearer by. In §3.4, we use the two values ℓ = 4 and 10 and the associated encounter-rate curves are displayed in figure 8. Each was generated by averaging the results of 10 000 simulations at each of 300 values for *κ*.

### 3.4. Placing model results in the context of disease ecology

In §2.2, we described our stochastic small-population model for pathogen invasion. We say that an invasion is ‘successful’ if it achieves a population level equivalent to what would be the endemic equilibrium of the deterministic version of the model. There exists an explicit formula for the probability of invasion, but it is difficult to interpret in terms of the parameters of the model. So, following Ball & Donnelly [42], we use an approximation for the true value (see equation (B 1) and further discussion in appendix B). This reduces our analysis to determining whether the total rate of transmission (which is affected by the resource-driven encounter rate) is greater than the disease-related mortality rate *ν*.

To assess whether a change in the consumer encounter rate is ‘large’ in the context of jackals and rabies, we followed Rhodes *et al.* [29] in establishing a background rate of pathogen transmission (*b* = 1 week^−1^) and a disease-related mortality rate (*ν* = 1.4 week^−1^) yielding the reproductive ratio 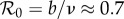. Since 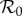, rabies is found to be subcritical.

**Figure 9.**
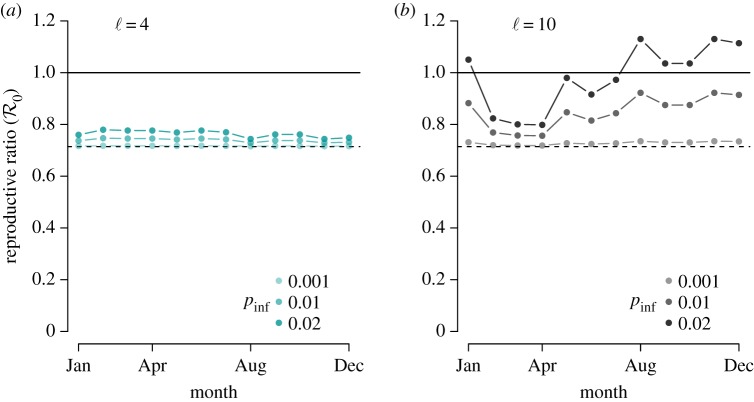
Disease model result incorporating encounter rate model simulations and empirical carcass density estimates from ENP. Time-dependent rabies reproductive ratio, as defined in equation (2.3), where the corresponding number of resource-driven encounters for each month is displayed in figure 8 as the intersection of the monthly resource density lines with the encounter curves. We used rabies parameters *b* = 1, *ν* = 1.4 and detection distances ℓ = 4 (*a*) or ℓ = 10 (*b*) as presented in table 1. Results are shown for three probability of infection values *p*_inf_ = 0.001, 0.01 and 0.02, where darker points represent higher probabilities. (Online version in colour.)

Using the month-by-month encounter rate values appearing in figure 8, which were generated using the parameter values displayed in table 1, we calculated the time-dependent reproductive ratio for six scenarios and displayed them in figure 9. Figure 9*a*,*b* corresponds to the distance of detection choices ℓ = 4 and ℓ = 10, respectively. In each case, we varied the probability of infection parameter *p*_inf_ to demonstrate its impact on the final result.

**Table 1.** Parameters used in the disease ecology analysis.

	value	units	definition	source
*b*	1	week^−1^	transmission rate	[29]
*ν*	1.4	week^−1^	rabies mortality rate	[23,29]
*κ*	0.005–0.043	km^−2^	carcass density	ENP data analysis
*ρ*	0.2	km^−2^	jackal territory density	[21,40,41]
ℓ	4–10	km	max distance of detection	ENP data analysis
*τ*	1	week	timescale of resource availability and visitation	ENP data analysis

When ℓ = 4, the resource density for each month is near or below the critical resource density κ_*_, i.e. the density for which the maximal encounter rate occurs. So an increase in resource density leads to an increase in the resource-driven encounter rate and resulting reproductive ratio, regardless of the *p*_inf_ value. However, because the peak of the encounter-rate curve is relatively low (approx. five per week, figure 8) the reproductive ratio, defined in equation (2.3), remains below the critical value of one (figure 9*a*). On the other hand, when ℓ = 10, most of the monthly resource densities are greater than κ_*_ (figure 8). In those cases, increases in resource density lead to decreases in the resource-driven encounter rate and resulting reproductive ratio. In this regime, we see that the months with low carcass availability are those most vulnerable to pathogen invasion (figure 9*b*). For ℓ < 4, we infer from figures 5 and 9*a* that the inclusion of resource-driven encounters will have minimal impact on the expected time-dependent reproductive ratio.

We note that the magnitude of change in 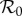 is directly dependent on the estimate for jackal territory density *ρ* (recall equation (3.1)). If *p*_inf_ = 0.02 and if *ρ* = 1 instead of *ρ* = 0.2, for example, the April 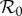 for ℓ = 4 would be (by way of combining (2.3) with (3.1)) approximately 1.07. A similar modification for ℓ = 10 would result in an August 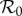 of 2.86. These results can readily be translated to a probability of successful invasion over the course of a resource increase of duration *T*. As described in §2.2 and appendix B, successful pathogen invasions arrive according to a Poisson process with rate *γ*_spillover_*p*_invasion_, where 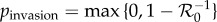. Assuming the transmission rate is constant over the period of interest, the probability of invasion is 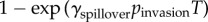.

## 4. Discussion

In this work, we have developed a framework for analysing the impact of changes in resource availability on the rate of conspecific encounters among consumers and express our results in the context of disease ecology. Given a landscape of consumers and resources, we essentially ask the question: would adding one more resource site lead to more or fewer encounters among the consumers?

We have proposed a novel consumer–resource interaction model to investigate this question. Through a combination of numerical simulation and mathematical analysis, we have identified and characterized two qualitatively distinct parameter regimes. In a low-resource regime, adding more resources leads to more consumer–consumer encounters; in a high-resource regime, adding more resources leads to fewer consumer–consumer encounters. In the high-resource regime, our model prediction is consistent with classical foraging theory. For example, in any model that results in an ideal free distribution of consumers, adding more resource patches should lead to less aggregation at resource sites, and therefore fewer encounters. For the low-resource regime though, our model results are dictated by the limited distance of detection, which has received less attention in the foraging literature.

The utility of our model is that it can be used to predict the qualitative dynamics of a system once certain fundamental parameters are estimated: the consumer density (*ρ*), the resource density (*κ*) and the maximum distance of detection and response (ℓ). The low- and high-resource regimes are separated by a peak in the number of encounters. One might expect that the critical resource density associated with the maximum number of encounters would depend on consumer density, but we find that it depends only on the maximum distance of detection: κ_*_ ≈ 0.5ℓ^−2^ (see equation (3.1), §3.1.)

To work through a specific case study, we used location data for a population of jackals and the carcasses upon which they scavenge in ENP. While some model parameters (*κ* and *ρ*) are fairly straightforward to estimate, others are not (see §3.3 for our approach to estimating the parameter ℓ in particular). One notable challenge that arises is that the definition of an ‘encounter’ is intrinsically subjective, depending strongly on the question of interest (also see Gurarie *et al.* [43] and White *et al.* [44] for further discussion on this point). To relate our resource-driven encounter rate to a rate of pathogen transmission from infectious to susceptible individuals, we introduced a corrective ‘probability of infection’ factor *p*_inf_. Because pathogen transmission is essentially impossible to directly observe, proper inference for such a parameter would likely require population-level disease incidence data that does not currently exist. In response to this uncertainty in parameter values, we display model results that emerge from a range of reasonable values for both *p*_inf_ and ℓ. The key takeaway is that for certain combinations of biologically relevant parameters, we confirm that small changes in the resource landscape can lead to substantial changes in pathogen transmission dynamics. In fact, our simulations show that a sudden scarcity of a resource can have a larger effect on encounter rates than a resource pulse (figure 5). In the context of ENP, this can be seen by comparing the model results for a month with high resource availability (April) to a month with low resource availability (August) in figures 8 and 9.

Building upon existing investigations into how changes in resource and consumer densities induce changes in disease dynamics, our work suggests that the relationship between territory size and the distance of resource detection plays a crucial role in determining infectious disease outcomes. To use the present context for an example, we note that jackals may use visual cues from vultures to identify carcass sites ([45] and anecdotal observations by an author and colleagues). If vulture populations decline, as has now been documented in both Asia and Africa [46], the detection distance for jackals could decrease, potentially causing a pathogen invasion regime shift. Interestingly though, the specific example of declining vulture populations exemplifies the complexity of consumer–resource interactions. In an experiment conducted by Ogada *et al.* [47], the authors found that there were increased encounters among mammalian scavengers when vultures could not see and react to carcasses (in contrast with figure 6*b*).

### 4.1. Opportunities for incorporating landscape heterogeneity

In order to study the effects of changing resource availability on encounter rates, we made some important simplifying assumptions about the consumer–resource landscape.

*Distribution of consumers.* Our model assumes that consumers are Poisson distributed across the landscape. We acknowledge that in reality the distribution of jackals and other territorial consumers might be under-dispersed relative to a Poisson model. Distributing consumers based on a model with lower variance would reduce the number of consumers located within the vicinity of any focal consumer and thus would reduce the resulting encounter rate. Qualitatively, this would not affect our encounter rate results since the reduction would be consistent across all parameter values considered. Relaxing this assumption in future work would inform the magnitude of reduction in encounters and have implications for whether the number of encounters supports a rabies reproduction number greater than one.

*Distribution of resources.* We assumed that the duration of resource availability and visitation, *τ*, was constant. Resources are made available to consumers simultaneously and the number of consumers does not affect how long a resource is viable for consumption. Variation in resource quality, geographical characteristics and local environmental factors can affect the model through multiple parameters. Resource sites that attract vultures might be detectable from larger distances than resources that do not, causing variation in ℓ. Small carcasses may be rapidly depleted, decreasing *τ*_1_, and may not satiate consumers, decreasing *τ*_2_. We might expect similar changes in the duration of resource availability and duration of satiation overall when carcasses are scarce. These changes in duration of availability can further influence the probability of infection term *p*_inf_. In terms of the selection algorithm, a consumer might not choose the closest available resource if one of greater quality is just a little bit farther away. Our model assumes uniformity in resources and, compared with predictions that would follow from each of these possible modifications, it produces a lower variance in the number of visitors to a given site. Future work could include an individual-based simulation that allowed resources to appear and disappear continuously in time based on how many consumers used them and the expected duration of availability.

### 4.2. Opportunities for integrating more detailed animal behaviour

The complex relationship between resource allocation, consumer behaviour and pathogen spread deserves further study. We constructed our model to be detailed enough to examine our primary question, but simple enough to permit rigorous mathematical analysis. While there are many ways to extend the model to account for more nuanced behaviour, we highlight a few.

*Resource detection and selection.* There are other natural models for the consumer's ability to detect resources, as well as for the algorithm determining which resource is visited, if any. For example, one could posit that there is imperfect detection and that the probability of detection decreases with a consumer's distance from the resource. Also, one could relax the restriction that the consumer always picks the closest detected resource. An informal investigation suggested that, as long as we pose assumptions consistent with those outlined at the beginning of §2.1, adopting alternative model specifications does not change the qualitative description of our results reported in §3.1. We opted for the version that yields the most explicit analytical results, but note that changes to model assumptions would likely change the value of the critical resource density κ_*_ as well as the height of the associated encounter rate peak. For example, our model could be adapted to allow jackals to visit multiple carcasses during a week. In that case, we would expect additional encounters and thus an increase in the magnitude of the encounter rate peak.

Additionally, incorporating social behaviours involved in detecting and selecting resources could have a qualitative impact on our results. It would be interesting to consider an alternate model where a consumer's choice to visit a resource was not independent from other consumers. Carcasses with many jackals present may be less desirable, due to increased competition for available flesh and possibly a heightened safety risk. However, it may be easier to detect carcasses when other jackals are already present at a carcass. Thus, it is not immediately clear whether we should expect encounters to increase or decrease in this scenario. Additionally, one could account for behaviour away from resource sites such as the tendency to avoid territorial conflicts when possible [23] and to travel efficiently (e.g. along roads instead of through thick brush). Moreover, it has recently been shown that heterogeneity in consumer personalities can be intimately tied to emergent population-scale properties like foraging efficiency and contact structure [11]. Including these factors is beyond the scope of this work, but might be crucial in specific applications.

*Modified behaviour of individuals.* In this theoretical investigation, we have assumed uniformity among individuals in order to focus on the effect of resource availability on encounter rates. However, heterogeneities between potential hosts and their contact rates have important implications for disease dynamics [48–50]. In the context of jackals, dispersing juveniles will likely have a higher than average expected encounter rate. Accordingly, they may contribute disproportionately to rabies spread.

Additionally, behavioural changes associated with the disease status of an individual may affect its expected encounter rate. Developing a theory for susceptible–infectious encounter rates that considers both types of individuals will be especially important for infections that alter host behaviour (e.g. rabies). Specifically, we note that the manner in which a rabid animal detects and selects resource sites could be much different than that of a susceptible individual. We leave the additional detail needed to provide a full characterization of rabies transmission dynamics for future exploration.

*Off-site encounters.* At present, our model considers the relationship between resource availability and the consumer encounter rate specifically at resource sites. However, a change in resource availability will also likely influence other types of encounters. For example, when consumers are forced to make long treks to scarce resources, they may be exposed to unfamiliar individuals. Distinguishing between typical encounters (e.g. with family members and territorial neighbours) and unique encounters with new individuals could be important for determining transmission dynamics [8,51].

*Dynamic population counts.* We considered a fixed population density (i.e. *ρ*, the jackal territory density). However, population sizes change on multiple timescales. Jackals have birth pulses that will change the local jackal population size on an annual basis (although, pups may not contribute substantially to pathogen spread). In the long term, consumer population size may respond to resource availability; when resources are abundant more consumers can be supported in the same area. This allows for smaller territories (increases in *ρ*). Specifically in Etosha, zebras and other ungulates are attracted to a grassland foraging area south of the salt pan. When compounded with anthrax outbreaks, it is possible that increased abundance of the highly desirable carcass food source in this area could help support an unusually dense jackal population. Considering figure 5 and equation (3.1), we see that if the consumer density varies with the resource density, then there are two competing effects: while increasing *κ* can decrease the number of encounters for fixed *ρ*, a simultaneously increasing *ρ* can overcome this effect. In order to fully characterize the effects of resource availability, future work could study encounter rates and population dynamics in tandem.

### 4.3. Concluding remarks

Substantial progress has been made in showing how consumer aggregations at resources can affect disease dynamics [17,18] and, separately, that uses data on contact networks to quantify heterogeneity in pathogen transmission [44,49]. However, to date these studies have not fully leveraged the surge in high-frequency animal movement data [52–54]. Here we show how, for a specific disease system, these data can inform estimation of the contact rate process, a critical component of pathogen transmission [55,56]. We highlight the importance of framing such empirical analyses within theory, identifying general principles that underpin the interaction between resources, movement and transmission.

## Data accessibility

The data used in this study are available on Movebank (movebank.org, study name ‘Black-backed jackal, Etosha National Park, Namibia’) and are published in the Movebank Data Repository with doi:10.5441/001/1.n1r7ds5r. Supplementary data are available from figshare: https://doi.org/10.6084/m9.figshare.5216113.v1 and https://doi.org/10.6084/m9.figshare.5216131.v1.

## Appendix A. Mathematical analysis of the resource-driven encounter model

The simplicity of the resource-driven encounter model invites a rigorous asymptotic analysis. More than demonstrating the non-monotone relationship between resource density and the consequent encounter rate in the consumer population, we can obtain the exponents of the power laws that govern the relationship.

In what follows (and in the main text) when we write *φ*(*x*) ∼ *x*^*α*^ as *x* → *a*, we mean that there exists some constant *C* ∈ (0, ∞) such that

For example, a result we will use below is that if *Y* ∼ Pois(*λ*) for some *λ* > 0, then  as *λ* → 0. This is because

and using the Taylor series expansion for the exponential (or simply L'Hôpital's rule), we have

A 1

For higher order terms, we will use Big-Oh notation: we say that *f*(*x*) = *O*(*g*(*x*)) near *x* = *a* if there exist constants *C* > 0 and *L* > 0 such that if |*x* − *a*| < *L*, then |*f*(*x*)| ≤ *C*|*g*(*x*)|.

As in the main text, *κ* and ℓ denote the resource intensity and maximum distance of detection, respectively. In the presentation of our results, we will assume that the consumer density *ρ* = 1. In appendix A.3, we will discuss how to modify the results when *ρ* ≠ 1.

We take the domain  to be a circle of radius *R* > 3ℓ centred at the origin. There is a *focal consumer* located exactly at the origin. Resources are distributed throughout  as a Poisson spatial process with intensity *κ*. Other, non-focal consumers are distributed throughout  as a Poisson spatial process with intensity one. Let ***x***_0_ = (0, 0) and enumerate the non-focal consumer locations {***x***_1_, …, ***x***_*N*_}, where . Furthermore, let ***z***_1_, …, ***z***_*Z*_ be the resource locations where . For each pair 1 ≤ *i* ≤ *N* and 1 ≤ *j* ≤ *Z*, let *δ*_*ij*_ : = |***x***_*i*_ − ***z***_*j*_|. For each *i* ∈ {0, …, *N*}, let . In other words, *η*_*i*_ is the index of the resource that is closest to the *i*th consumer. For notational expediency, we will write the index of the resource closest to the focal consumer, *η*_0_, to simply be *η*.

In the above notation, we can express *β*, the number of resource-driven encounters experienced by the focal consumer, to be

A 2

Given a set of resource locations, it is useful to think of the landscape partitioned according to the associated Voronoi tessallation. That is, neglecting a set of measure zero,

We say that  is a *basin of attraction* for resource *i*: all consumers located in  will choose resource *i* as their resource to visit if it is within their detection radius. We define *B*(***x***; *r*) to be the circle of radius *r* centred at the location ***x***. Then the distribution of the encounter variable *β* conditioned on a given resource landscape  is

A 3

where we recall that *η* is the index of the resource chosen by the focal consumer and  if the event *A* occurs, and is zero otherwise.

**A.1. Small resource density and/or small detection distance**

Theorem A.1. *Let*  *be defined as in* (A 2)*. Then*  *and*  *as* *κ* *and* ℓ *go to zero, respectively. To be precise*,

A 4

Proof. We first introduce some notation. Let *N*(*r*) and *Z*(*r*) denote the number of consumers and resources within a distance *r* of the focal consumer. We proceed by conditioning on the number of resources that are near the focal consumer. We partition the sample space *Ω* as follows:

Naturally, it follows that  and we will find that the dominant term is the one associated with *Ω*_10_. Looking at the other terms, first observe that  since, if there are no resources to consume, the focal consumer will not have any encounters. To deal with the event *Ω*_2_, we apply equation (A 1) above, noting that the number of resources within detection distance of the focal consumer has the distribution *Z*(ℓ) ∼ Pois (*κπ*ℓ^2^). It follows that for small *κ*,  and for small ℓ, . To bound the conditional expectation , observe that the number of resource-driven encounters experienced by the focal consumer must be less than or equal to the number of consumers that are located within a distance of ℓ of the focal resource (the resource chosen by the focal consumer). Because this is a region of size *π*ℓ^2^, we have . Together we have that

For the event *Ω*_11_ we again exploit that, when the detection distance or resource density is small, it is unlikely that there will be more than one resource near the focal consumer. By independence of the resource distribution in disjoint regions

since the area of the annulus covering the region that is between a distance of ℓ and 3ℓ of the origin is 8*π*ℓ^2^. It follows that . Meanwhile, using the same upper bound on the number of resource-driven encounters experienced by the focal consumer, we have . Therefore,

Turning our attention to the event *Ω*_10_, if there is only one resource in the focal consumer's detection radius, and the resource is the only one in the larger 3ℓ radius circle centred at the origin, then all consumers within a radius ℓ of the focal resource will choose the same resource as the focal consumer. In other words, the number of encounters conditioned on *Ω*_10_ is *β*|_*Ω*_10__ ∼ Pois(*π*ℓ^2^). What was an upper bound in previous cases is now equality. It follows that . To compute the event's probability we argue as before,

A 5

Therefore,

and

as claimed. ▪

**A.2. Analysis in the high resource and large distance of detection regimes**

In the high resource density and large distance of detection regime, we are unable to get exact results. This is due to a fundamental barrier in the analysis that we will describe below. In the high-density regime, we can provide what appears to be a lower bound on  that, from the numerics, seems to scale with  as *κ* → ∞.

Our conjecture is based on the following heuristic. Recall that  is the basin of attraction that contains the focal consumer. Then

— Conditioned on the landscape of resources, the number of encounters experienced by the focal consumer is Poisson distributed with mean equal to its containing basin of attraction. Therefore, .
— *Unconfirmed estimate*: .
—  as *κ* → ∞.

The third part of the heuristic is established by lemma A.4 below. The second part of the heuristic is justified by the following.

Lemma A.2. *Let a resource landscape be given as described above and let the total region*  *be partitioned according to a Voronoi diagram generated using the resource locations* {***z***_1_, …, ***z***_*Z*_}. *We denote the areas of each of these basins of attraction* {*A*_1_, …, *A*_*Z*_}. *Let*  *be a random location in the landscape and define* *η* *to be the index of the basin of attraction that contains this point. Then*

Remark A.3. Unfortunately, at this time, we are not able to extend the result to establish the claim that the basin of attraction specifically containing the origin has an expected area that is larger than . Numerics strongly support this conclusion.

Proof.

where, in the last line, we have used the Cauchy–Schwarz inequality. ▪

Lemma A.4. *Suppose Y* ∼ *Pois*(*λ*). *Then*

A 6

Proof. Recall the exponential integral function

for *x* > 0, where the integral is taken in the sense of the Cauchy principal value. The exponential integral function can be written in terms of the series [57].

For large *x*, Ei(*x*) has the asymptotic expansion [58],

A 7

Following the suggestion of Grab & Savage [59], we note that

A 8

where *γ* is the Euler–Mascheroni constant. Combining (A 7) and (A 8), we arrive at the desired result. ▪

There is a fundamental mathematical barrier to making more progress on this problem. The preceding analysis reduces the problem to analysing the distribution of areas of cells generated by Poisson Voronoi tessellations, but this an outstanding mathematical problem [32]. In particular, there is no known expression for , the expected area of the basin of attraction that contains the focal consumer. This prevents us from obtaining a result for the large distance of detection regime that is explicit in *κ*.

Theorem A.5. *is an increasing function in* ℓ *and*

A 9

Proof. For a given *κ* > 0, let  denote a landscape of resources generated by a spatial Poisson process with intensity *κ*. For each such landscape, let  be the number of encounters for the focal consumer. As noted above in equation (A 3), this is the number of consumers located in a radius ℓ of the focal resource multiplied by one or zero depending on whether the focal resource is within radius ℓ of the focal consumer. For a fixed landscape, note that

As such,

Because this holds for all , the proposition follows. ▪

**A.3. Converting results for non-unit consumer density**

All of the preceding results have been expressed under the consumer density assumption *ρ* = 1. Similarly all simulations were conducted with *ρ* = 1. Extending the earlier notation, let  be the expected number of encounters for the focal consumer for the given triplet of parameters. We claim that, although there are three fundamental parameters in the model, there are only two degrees of freedom in the parameter space. That is to say, given a triplet (*ρ*, *κ*, ℓ) there exists a unique pair  such that . Namely,

To see this, let *N*(3ℓ) and  denote the number of consumers in the model within three times the maximum distance of detection for the parameter triplets (*ρ*, *κ*, ℓ) and , respectively. Let *Z*(3ℓ) and  denote the same for resources. Because consumers and resources are distributed as Poisson spatial processes, these values completely define the system. Furthermore, because Poisson random variables are completely parametrized by their means, it follows that  if  and . For the first constraint,

from which it follows that . Meanwhile

meaning that .

We note that this reduction of the problem to two parameters amounts to a non-dimensionalization of the three parameter model. The units of *ρ* and *κ* are both [length]^−2^, while ℓ has units of [length]. As a result,  and  are both dimensionless.

Revisiting the theorems of the previous sections we have the results:

A 10

and

A 11

## Appendix B. Mathematical analysis of the pathogen invasion model

Just as it is for the ODE SIS model, the reproductive ratio is a critical dimensionless parameter in the stochastic SIS model. When , then as *N* → ∞, *p*_invasion_ → 0 [60]. On the other hand, when , then as *N* → ∞, the probability of invasion is strictly greater than zero. There exist exact solutions to this hitting probability problem, however, such a presentation makes it difficult to understand how *p*_invasion_ depends on *b* and *ν*. Therefore, *p*_invasion_ is commonly approximated by computing the complement of an extinction probability for an associated branching process [42]. In terms of our CTMC model, this is equivalent to introducing the assumption that the size of the susceptible pool is very large with respect to the initial infectious population, and setting (*N* − *I*)/*N* ≈ 1 [61]. The modified rate functions for the CTMC are linear and take the form

The infectious population process is then a Galton–Watson branching process. The only two outcomes for such a process are extinction or explosion to infinity. The analysis reduces to recasting the CTMC as a discrete time generation-by-generation branching process that is defined in terms of the *offspring distribution*, i.e. the distribution of the number of offspring an individual might have before dying. In our case, the ‘offspring’ are the infections spawned by a single individual. Since the infection events occur according to a Poisson process with rate parameter *b* and the death of the individual occurs at rate *ν*, the number of successful infections before death is Geometrically distributed with success probability *b*/(*b* + *ν*). One can then show that, under the assumption that *b* > *ν*,

If *b* ≤ *ν*, the process goes extinct with probability one. It is from this calculation, and our formula for the basic reproductive ratio that we adopt the approximation

A 12

With this in hand, we can approximate the probability that there is a successful pathogen invasion in the target population during the period of increased resource availability, *t* ∈ [0, *T*]. For each pathogen introduction, we label it ‘successful’ with probability *p*_invasion_ and label it ‘unsuccessful’ otherwise. According to standard Markov chain theory [62] the arrival of *successful* introductions is a ‘thinned’ Poisson process, with arrival rate *γ*_spillover_*p*_invasion_. Intuitively, this means that the time it takes for a successful invasion is scaled by a factor of *p*_invasion_ in terms of the ‘rate of arrival’. It follows that the time of the arrival of the first successful spillover event has an exponential distribution with rate parameter *γ*_spillover_*p*_invasion_. Therefore, the probability of a successful invasion occurring during a resource pulse of length *T* has the form 1 − e^−*Tγ*_spillover_^^*p*_invasion_^.
